# Changes in RDW according to prognostic predictors in newly diagnosed multiple myeloma

**DOI:** 10.1038/s41598-024-53385-6

**Published:** 2024-02-03

**Authors:** Melania Carlisi, R. Lo Presti, F. Plano, S. Mancuso, S. Siragusa, G. Caimi

**Affiliations:** 1 Department of Health Promotion, Mother and Child Care, Internal Medicine and Medical Specialties, University of Palermo, Via del Vespro 129, 90127 Palermo, Italy; 2 Department of Psychology, Educational Science and Human Movement, University of Palermo, Palermo, Italy

**Keywords:** Cancer, Medical research, Oncology, Risk factors

## Abstract

RDW is an erythrocyte index that increase in multiple myeloma, in which it appears to have an important role in predicting outcome. For this reason, we performed a retrospective analysis to evaluate the relationships of RDW with some important prognostic predictors. Specifically, in a cohort of 190 newly diagnosed multiple myeloma patients, we have examined the behaviour of RDW and its trend in relation to the ISS stage and other prognostic factors, such as albumin, beta-2 microglobulin, LDH and bone marrow plasma cell infiltration. We performed the analysis in the entire cohort of patients and in the three different disease isotypes (Light chain, IgA, and IgG multiple myeloma). The evaluation of RDW in the different isotypes was made with the Kruskal–Wallis test, integrated by the Dunn test. The comparison between the subgroups allocated above and below the median value of each prognostic factor, was made with the Mann–Whitney test. From our analysis, we observed that RDW is higher in the IgA multiple myeloma, and it increases significantly from ISS I to III. Moreover, RDW increases in the presence of lower albumin values, higher levels of beta2-microglobulin and LDH and in the presence of a greater bone marrow plasma cell infiltrate.

## Introduction

Erythropoiesis is the process by which the proliferative and differentiated activity of the erythroid progenitors produces 2.5 × 10^11^ red blood cells daily. For a long time now, the evaluation of peripheral blood count has been entrusted to automated blood counters, reaching a higher level of precision than manual counting. Among the blood count parameters, the red cell distribution width (RDW) is of particular significance, favouring a complete evaluation of the erythrocyte component. RDW is a dimensionless quantitative parameter that represents the standard deviation of red blood cell volume divided by the mean volume, and it reflects the variation in cell size in the erythrocyte population. In different clinical situations it is possible to observe an increase in RDW and the main causes lie in one of these four possibilities: decreased of erythrocyte mean volume, increased reticulocyte volume variance, increased heterogeneity in the rate of RBC volume occurring in the peripheral circulation, and delayed RBC clearance^1^. Furthermore, any change in RDW should be assessed considering haemoglobin values, erythrocyte number and mean corpuscular volume, respectively.

The RDW increase is mainly found in microcytic and macrocytic anemia, anemia from chronic inflammatory diseases, haemoglobinopathies and thalassemic syndromes, in microangiopathic haemolytic anemia and disseminated intravascular coagulopathy. However, in recent years it also been observed in acute myeloid leukemia^2^, chronic myeloid leukemia^3–5^, in myelodysplastic syndromes^6–8^, in primary myelofibrosis^9^, polycythemia vera and essential thrombocythemia^10–12^ and especially in multiple myeloma (MM)^13^.

MM is a malignant hematological neoplasm characterized by clonal proliferation of plasma cells that can release monoclonal immunoglobulins or fractions of them. MM accounts for 1.8% of all new cancer cases. The rate of new cases of myeloma was 7.1 per 100,000 men and women per year, and it appears to have at 5-year relative survival rate of 58.9%^14^. In the first research concerning the behaviour of RDW in multiple myeloma^15^ it was concluded that the increase in this erythrocyte index was associated with the advanced stage of disease and poor prognosis. Subsequently, other authors confirmed the role of RDW in predicting outcome of MM patients, in short- and long-distance^16,17^, and still others hypothesized a role of this erythrocyte index in prediction of clinical response to treatment^18,19^. Moreover, a recent systemic review and a meta-analysis of all publications regarding the trend of RDW in multiple myeloma was carried out, with the conclusion that this index is a significant predictor of outcomes in new diagnosed multiple myeloma patients^20^.

Considering therefore the role of RDW in MM, it has meanwhile begun to analyse the possible causes responsible for the significant variation of this index. Several literature data are available about the relations between RDW and inflammatory markers, such as erythrocyte sedimentation ratio and C-reactive protein^21^ but, in MM, is more plausible than the variation of RDW may mainly depend on high circulating levels of interleukin-6 (IL-6), tumor necrosis factor-alpha (TNF-alpha) and hepcidin^22–24^. Interacting with each other, these factors tend to alter the erythropoiesis, causing significant variation in the erythrocyte maturation and, inevitably, contributing to the increase of the RDW. In this regard, it should also be noted that the IL-6 is a key factor in the survival and proliferation of myeloma cells, and, at the same time, it is a marker of the negative trend of the disease^25^. Moreover, the myeloma cells have high cellular levels of reactive oxygen species and low levels of antioxidant molecules, both related to the increased oncogenic activity and amplified metabolic action^26^. Beyond the altered cytokine network present in MM^27^ there seems to be also an altered balance between the inflammatory and anti-inflammatory pattern, and between the proliferative and anti-proliferative one^28^.

Considering the above, in this single-centre retrospective analysis, we considered the RDW in a cohort of 190 patients with newly diagnosed symptomatic MM before systematic treatment, to examine the trend of this erythrocyte index in relation to the various MM isotypes, to the International Staging System (ISS) and, especially, to the main prognostic predictors (albuminemia, beta-2 microglobulin, LDH and plasma cell infiltration in the bone marrow).

## Results

A total of 190 patients were eligible for this analysis. The median age was 69 ± 10 years, and 88 (46.3%) were male. 107 patients had a diagnosis of IgG MM (71 IgG κ and 36 IgG λ), 56 patients IgA MM (28 IgA κ and 28 IgA λ isotype) and 27 patients had a diagnosis of light chain multiple myeloma (LCMM), of which 13 with expression of κ light chain and 14 with λ light chain. Regarding the stage of disease, we observed an ISS I in 22% of patients (41/190), ISS II in 26% (49/190) and ISS III in 52% of whole cohort (100/190). The characteristics of the patients are presented in Table 1.

**Table 1:** Characteristics of patients.

Parameters	Mean/percentage
Sex	
(Male)	46% (88/190)
(Female)	54% (102/190)
Age at diagnosis	69 ± 10
ISS stage	
Stage I	22% (41/190)
Stage II	26% (49/190)
Stage III	52% (100/190)
Isotype	
IgA k	15% (28/190)
IgA λ	15% (28/190)
IgG k	37% (71/190)
IgG λ	20% (36/190)
Light chain k	6% (13/190)
Light chain λ	7% (14/190)
LDH U/L (normal range 50–250)	193 ± 91
Serum calcium mg/dL (normal range 8.6–10.21)	9.57 ± 1.03
Serum creatinine mg/dL (normal range 0.51–0.95)	1.55 ± 1.58
Monoclonal Component g/L	26.01 ± 20.42
Serum albumin g/L (normal range 35–52)	35.1 ± 6.39
β-2 Microglobulin mg/L (normal range 0.8–2.2)	7.50 ± 7.78
Bone marrow plasma cells (%)	45 ± 26
Hemoglobin g/dL (normal range 12–16 l)	11.11 ± 6.14
Hematocrit % (normal range 35–48)	32 ± 5.70
MCV fL (normal range 80–99)	91.83 ± 8.36
RDW-CV% (normal range 11–15)	15.77 ± 2.68

After evaluating the medians, interquartile intervals and the range of the parameters considered (hematocrit, haemoglobin, mean corpuscular volume and RDW) in the whole cohort of MM patients (Supplementary Table 1), we compared each parameter in the three different MM isotypes (Table 2), finding that only the RDW seems to have a discriminating role; in fact, its value is significantly higher in the IgA MM respect to IgG e light chain MM (LCMM). Subsequently, we investigated the behaviour of each parameter in relation to the different ISS stage, observing a reduction in hematocrit and in hemoglobin, and an increase in RDW proceeding from stage I to stage III, without any change in the mean corpuscular volume (Table 3). At this point, we analyzed the medians of some MM prognostic predictors, such as albumin, beta-2 microglobulin, LDH and percentage of bone marrow plasma cell infiltration (BMPC), both in whole cohort and in the three different isotypes, and therefore, we examined the trend of each parameter in relation to the median of singly prognostic predictor (Supplementary Table 2).

**Table 2:** Medians (IQRs) of the erythrocyte parameters in MM patients subdivided according to the monoclonal protein.

	LCMM (n = 27)	IgA MM (n = 56)	IgG MM (n = 107)	KWS	*p*
Ht %	31.40 (11.70)	30.35 (8.42)	31.90 (8.80)	0.165	0.4285
Hb (g/dL)	10.50 (4.1)	9.95 (2.65)	10.30 (3.20)	3.122	0.2100
MCV (fL)	92.90 (10.6)	92.90 (8.22)	92.40 (9.20)	0.8236	0.6625
RDW %	14.40 (3.10)	16.00 (4.40)*	14.80 (3.10)^#^	9.911	0.0070

*IQR* interquartile range, *MM* multiple myeloma, *LCMM* light chain multiple myeloma, *KWS* Kruskal–Wallis statistic, *Ht* haematocrit, *Hb* hemoglobin, *MCV* mean cell volume, *RDW* red blood cells distribution width.

*p < 0.05 vs LCMM (Dunn’s multiple comparison test).

^#^p < 0.05 vs IgA MM (Dunn’s multiple comparison test).

**Table 3:** Medians (IQRs) of the erythrocyte parameters in MM patients subdivided according to the ISS stage.

	ISS stage I (n = 41)	ISS stage II (n = 49)	ISS stage III (n = 100)	KWS	*p*
Ht %	36.70 (9.45)	33.00 (6.90)	29.45 (6.87)***^#^	24.63	< 0.0001
Hb (g/dL)	12.10 (3.60)	10.50 (2.50)*	9.65 (2.20)***^#^	28.66	< 0.0001
MCV (fL)	92.40 (9.55)	93.20 (10.9)	92.25 (8.22)	1.487	0.4755
RDW %	14.00 (2.10)	14.60 (2.25)	15.90 (3.77)***	15.48	0.0004

*IQR* interquartile range, *MM* multiple myeloma, *ISS* international staging system, *KWS* Kruskal–Wallis statistic, *Ht* haematocrit, *Hb* haemoglobin, *MCV* mean cell volume, *RDW* red blood cells distribution width.

*p < 0.05 ,***p < 0.001 vs ISS stage I (Dunn’s multiple comparison test).

^#^p < 0.05 vs ISS stage II (Dunn’s multiple comparison test).

Regarding albumin levels, in the whole cohort of patients we observed, for values below the median, a significant reduction in hematocrit and hemoglobin, associated with a slight increase in the mean corpuscular volume and, above all, a noticeable increase in RDW (Table 4A). The same analysis was carried out in the LCMM showing a reduction in hemoglobin level only (Table 4B). In IgA MM (Table 4C) and IgG MM (Table 4D), the subdivision for albumin levels shows a decrease in hematocrit and hemoglobin associated with a marked increase in RDW, for values below the respective medians.

**Table 4:** Medians (IQR) of the erythrocyte parameters according to the albumin in MM and their isotypes.

A) All MM (n = 190)	Albumin < median (n = 94)	Albumin ≥ median (n = 96)	*p*
Ht %	29.45 (7.80)	32.90 (8.80)	< 0.0001
Hb (g/dL)	9.60 (2.23)	11.00 (3.00)	< 0.0001
MCV (fL)	93.90 (8.23)	91.65 (8.87)	0.0494
RDW %	16.10 (3.67)	14.35 (2.27)	< 0.0001

B) LCMM (n = 27)	Albumin < median (n = 12)	Albumin ≥ median (n = 15)	*p*
Ht %	30.50 (6.75)	37.80 (12.80)	0.1001
Hb (g/dL)	10.30 (1.98)	12.20 (4.80)	0.0764
MCV (fL)	94.00 (17.30)	90.90 (9.90)	0.3994
RDW %	15.10 (6.15)	14.20 (1.90)	0.2717

C) IgA MM (n = 56)	Albumin < median (n = 28)	Albumin ≥ median (n = 28)	*p*
Ht %	29.05 (8.75)	31.40 (8.70)	0.0320
Hb (g/dL)	9.75 (2.88)	10.30 (3.105)	0.0573
MCV (fL)	93.60 (7.50)	90.55 (9.67)	0.4131
RDW %	17.00 (3.45)	14.40 (3.72)	0.0042

D) IgG MM (n = 107)	Albumin < median (n = 53)	Albumin ≥ median (n = 54)	*p*
Ht %	29.10 (7.15)	33.85 (7.33)	< 0.0001
Hb (g/dL)	9.60 (2.45)	11.05 (2.68)	0.0001
MCV (fL)	94.00 (8.60)	91.75 (9.48)	0.2308
RDW %	15.90 (3.80)	14.35 (1.88)	0.0012

*IQR* interquartile range, *MM* multiple myeloma, *Ht* haematocrit, *Hb* haemoglobin, *MCV* mean cell volume, *RDW* red blood cells distribution width.

In relation to the beta-2 microglobulin levels, in the entire study population, a reduction of hematocrit and hemoglobin and a marked increase in RDW were associated with values exceeding the median; no change was recorded for the mean corpuscular volume (Table 5A). The same evaluation was carried out in LCMM observing a decrease in hematocrit and hemoglobin (Table 5B). In IgA MM (Table 5C) the subdivision according to beta-2 microglobulin levels has recorded, for values exceeding the median, a reduction of hemoglobin with an increase in RDW, while in IgG MM (Table 5D) the same analysis has highlighted a decrease in hematocrit and hemoglobin associated with an increase in RDW.

**Table 5:** Medians (IQR) of the erythrocyte parameters according to the Beta2-MG in MM and their isotypes.

A) All MM (n = 190)	Beta2-MG < median (n = 94)	Beta2-MG ≥ median (n = 96)	*p*
Ht %	33.95 (9.05)	29.55 (6.78)	< 0.0001
Hb (g/dL)	11.10 (3.055)	9.65 (2.18)	< 0.0001
MCV (fL)	92.90 (9.68)	91.65 (8.15)	0.6895
RDW %	14.40 (2.72)	15.90 (3.45)	0.0006

B) LCMM (n = 27)	Beta2-MG < median (n = 13)	Beta2-MG ≥ median (n = 14)	*p*
Ht %	38.30 (9.90)	27.90 (8.37)	0.0041
Hb (g/dL)	13.30 (3.65)	9.70 (2.005)	0.0053
MCV (fL)	93.00 (11.80)	89.95 (12.28)	0.8300
RDW %	13.50 (5.75)	14.55 (2.03)	0.8020

C) IgA MM (n = 56)	Beta2-MG < median (n = 28)	Beta2-MG ≥ median (n = 28)	*p*
Ht %	32.85 (6.95)	29.60 (8.70)	0.1900
Hb (g/dL)	10.40 (2.575)	9.55 (2.605)	0.0552
MCV (fL)	93.95 (9.35)	90.35 (7.95)	0.6993
RDW %	15.10 (3.20)	17.20 (5.07)	0.0097

D) IgG MM (n = 107)	Beta2-MG < median (n = 53)	Beta2-MG ≥ median (n = 54)	*p*
Ht %	34.50 (7.80)	29.65 (5.50)	0.0003
Hb (g/dL)	11.40 (2.85)	9.75 (2.20)	0.0001
MCV (fL)	92.40 (9.50)	92.15 (8.27)	0.3395
RDW %	14.10 (2.25)	15.70 (2.67)	0.0061

*IQR* interquartile range, *MM* multiple myeloma, *Beta2-MG* Beta2-microglobulin, *Ht* haematocrit, *Hb* haemoglobin, *MCV* mean cell volume, *RDW* red blood cells distribution width.

Relative to LDH levels, in the whole cohort of patients, in presence of values above the median, we observed a decrease in hematocrit and an increase in RDW, while no change was recorded for hemoglobin values and mean corpuscular volume (Table 6A). The same evaluation performed in LCMM did not reveal any significant variation of erythrocyte indexes for values below and above the median (Table 6B). In IgA MM (Table 6C), for values exceeding the median, we found a reduction in hematocrit and hemoglobin associated with an increase in RDW, while in IgG MM (Table 6D) the same approach has highlighted, in the subgroup with values beyond the median, an increase in RDW only.

**Table 6:** Medians (IQR) of the erythrocyte parameters according to the LDH in MM and their isotypes.

A) All MM (n = 190)	LDH < median (n = 95)	LDH ≥ median (n = 95)	*p*
Ht %	33.00 (9.40)	30.40 (9.20)	0.0409
Hb (g/dL)	11.00 (3.00)	10.00 (2.40)	0.3290
MCV (fL)	92.40 (8.20)	92.90 (9.70)	0.9795
RDW %	14.60 (2.70)	15.80 (4.40)	0.0014

B) LCMM (n = 27)	LDH < median (n = 13)	LDH ≥ median (n = 14)	*p*
Ht %	33.20 (12.35)	31.35 (12.50)	0.9526
Hb (g/dL)	10.50 (4.20)	10.55 (4.55)	0.7835
MCV (fL)	90.90 (13.05)	93.35 (11.85)	0.9430
RDW %	14.50 (2.70)	14.30 (4.85)	0.8020

C) IgA MM (n = 56)	LDH < median (n = 28)	LDH ≥ median (n = 28)	*p*
Ht %	33.40 (11.40)	29.95 (6.93)	0.0655
Hb (g/dL)	11.05 (3.025)	9.75 (1.85)	0.0216
MCV (fL)	93.15 (5.65)	92.05 (11.13)	0.7916
RDW %	15.30 (3.05)	17.50 (4.83)	0.0288

D) IgG MM (n = 107)	LDH < median (n = 53)	LDH ≥ median (n = 54)	*p*
Ht %	32.70 (7.85)	30.85 (9.65)	0.2872
Hb (g/dL)	11.00 (2.90)	10.25 (3.05)	0.2117
MCV (fL)	92.40 (8.50)	91.00 (9.73)	0.5564
RDW %	14.60 (2.35)	15.60 (3.15)	0.0100

*IQR* interquartile range, *MM* multiple myeloma, *LDH* lactate dehydrogenase, *Ht* haematocrit, *Hb* haemoglobin, *MCV* mean cell volume, *RDW* red blood cells distribution width.

Finally, regarding bone marrow plasma cell infiltration, in the whole study group (Table 7A), for values above the median, a reduction in hematocrit and hemoglobin associated with an increase in RDW was evident. Performing the same analysis in LCMM (Table 7B) and IgA MM (Table 7C), we did not observe any difference. While, in IgG MM (Table 7D), for values exceeding the median, a reduction in hematocrit and hemoglobin, accompanied by an increase in RDW was observed.

**Table 7:** Medians (IQR) of the erythrocyte parameters according to the BMPC% in MM and their isotypes.

A) All MM (n = 190)	BMPC% < median (n = 92)	BMPC% ≥ median (n = 98)	*p*
Ht %	32.70 (9.50)	30.25 (8.35)	0.0024
Hb (g/dL)	10.90 (3.375)	10.00 (2.63)	0.0031
MCV (fL)	92.10 (9.95)	92.90 (8.47)	0.8224
RDW %	14.60 (3.23)	15.65 (3.63)	0.0035

B) LCMM (n = 27)	BMPC% < median (n = 11)	BMPC% ≥ median (n = 16)	*p*
Ht %	33.20 (12.70)	30.50 (11.95)	0.2261
Hb (g/dL)	10.50 (5.00)	10.35 (3.00)	0.4006
MCV (fL)	86.90 (10.20)	94.20 (10.95)	0.2720
RDW %	14.00 (2.90)	14.60 (3.97)	0.2997

C) IgA MM (n = 56)	BMPC% < median (n = 26)	BMPC% ≥ median (n = 30)	*p*
Ht %	30.35 (9.10)	30.65 (8.77)	0.5166
Hb (g/dL)	9.95 (2.655)	10.05 (3.155)	0.7473
MCV (fL)	92.90 (8.22)	93.35 (9.00)	0.7610
RDW %	16.00 (4.10)	16.00 (4.70)	0.6865

D) IgG MM (n = 107)	BMPC% < median (n = 49)	BMPC% ≥ median (n = 58)	*p*
Ht %	32.70 (8.45)	30.85 (7.4)	0.0615
Hb (g/dL)	11.00 (3.10)	10.20 (2.605)	0.0273
MCV (fL)	92.40 (10.95)	91.95 (8.83)	0.8653
RDW %	14.00 (2.80)	15.55 (2.68)	0.0031

*IQR* interquartile range, *MM* multiple myeloma, *BMPC* bone marrow plasma cell, *Ht* haematocrit, *Hb* haemoglobin, *MCV* mean cell volume, *RDW* red blood cells distribution width.

## Discussion

The first finding that emerges from our analysis is that RDW, when evaluated in the three different MM isotypes, is higher in IgA MM, without any variation in the remaining considered parameters. From the literature data, RDW not appear to be related to the marked cytogenetic abnormalities or the worse prognosis that characterize this MM isotype^29–33^; rather, in IgA MM, notoriously associated with a worse prognosis, the increase in RDW could be related to an evident reduction, although not significant, in hematocrit and hemoglobin values, with a negative correlation of RDW with each of these two parameters (data not shown).

After this first consideration, in the rest of the discussion and in the concluding remarks, we would like to give priority to the data concerning the RDW in the whole study group respect to the specific MM isotypes, especially considering the different and not uniformly distributed number of patients into the three isotypes.

Interesting is the trend of the RDW proceeding from ISS stage I to III; in this evaluation, as is to be expected, the increase in RDW is associated with a parallel progressive decline in hemoglobin values.

Dividing the whole cohort of patients according to serum albumin levels, fundamental parameter for calculating the ISS, it is evident that, for values below the median, there is a clear reduction in hematocrit and hemoglobin, and especially an increase in RDW. It has long been established that the reduction of albumin accompanies MM^34^ and, at the same time, it is documented as the reduced hepatic synthesis of albumin is to be related to the increase of interleukin-6 (IL-6) and to the altered cytokine network^27,35^. In fact, the altered cytokine profile reduces the daily hepatic synthesis of albumin that, even if dynamic, seems to attest itself on values of 200 mg per kilogram of body weight^36^. Numerous data in the literature underline the prognostic value of albumin levels in patients diagnosed with multiple myeloma^37^ and, recently, from an analysis performed on 2377 MM patients, the reduced serum albumin levels have been qualified, once again, as predictors of early mortality (less than 12 months)^38^.

Evaluating the patients according to the levels of beta-2 microglobulin, another important parameter which, together with albumin, is used to calculate the ISS, we observe, in the subgroup with values above the median, a decrease in hematocrit and hemoglobin associated with the increase in RDW. Beta-2 microglobulin is the light chain of histocompatibility antigen (HLA) and it is expressed on the surface of several nucleated cells, but, when these cells undergo a neoplastic transformation, it moves from the intracellular to the extracellular compartment. It is not a specific marker, but it increases in lymphoprolipherative diseases including multiple myeloma^39,40^. When the kidney function is normal, the beta-2 microglobulin seems to reflect the entire mass of myeloma cells and, in our study population, as expected, it is negatively related to albumin levels (p < 0.001).

Always in the whole patient group, the subdivision carried out according to the LDH levels has recorded a reduction in hematocrit and an increase in RDW in the subgroup with values beyond the median. The LDH is expression of impaired cellular metabolism and its prognostic value in MM is has long been known. In fact, together with cytogenetic data, LDH is used for the calculation of the revised-ISS (R-ISS), and it has been also indicated as a marker of chemoresistance and therapeutic refractoriness^41–44^. In a recent analysis performed on 859 newly diagnosed MM patients and staged in accordance with the Revised International Staging System (R-ISS), it has found that high levels of LDH were independent predictors of a worse prognosis^45^; in addition, evaluating the overall survival of 502 newly diagnosed MM patients stratified according to the frequent and complex genetic abnormalities not included in the R-ISS it underscored the undisputed role of high levels of this enzyme^46^.

Finally, dividing the study population according to BMPC infiltration, in the subgroup with a percentage above the median value, it is evident a reduction in hematocrit and hemoglobin associated with an increase in RDW. The BMPC infiltration has a key role in multiple myeloma; in fact, not only it correlates with the risk of progression in smoldering multiple myeloma and monoclonal gammopathy of undetermined significance but, in symptomatic multiple myeloma, a plasma cell infiltrate > 60% represents a specific treatment criteria, and, at now, it is included among the most recent SLiM CRAB criteria^47,48^. In fact, Initially, a percentage lower than 50% was in fact considered indicative of a better prognosis^49^ but, as emerges from a more recent study conducted on 1426 patients, the threshold is now represented by 60%, with lower values considered predictive of overall and progression free survival^50^.

The attention paid to the RDW has not been occasional at all; in fact, in addition to the important prognostic role in MM, in the last decade it has been also investigated a possible correlation between RDW and red cell deformability. This topic has been evaluated in subjects with any pathology^51^, in hypertensive patients^52^, in acute myocardial infarction^53^, in patients with metabolic diseases and thalassemic trait^54^ and, recently, also in patients with haematological diseases^55^. However, while in healthy subjects and in patients with hematological neoplasms the correlation between these two parameters was significantly negative, in other clinical disorders the same correlation was positive and not easy to interpret. In our study population, represented by 190 patients with newly diagnosed MM, we do not have any data on erythrocyte deformability, but previously, although in a limited number of MM patients, we have highlighted a reduction of erythrocyte deformability with diffractometric technique^56–60^. So far, we have charged this alteration to anomalies of the dynamic properties of the membrane, and particularly, to its lipid composition and to the behaviour of phosphatidylserine that tend to move from the inner to the outer leaflet. In fact, in MM, up to now, the attention has been focused on the membrane and not on other components of red cell deformability such as the surface/volume ratio and internal viscosity affected by cytosolic calcium, by organic phosphates and by changes in hemoglobin.

In conclusion, in MM the RDW is not only increased, but is significantly related to the ISS stages and the main prognostic predictors, such as serum levels of albumin, beta-2 microglobulin, LDH and bone marrow plasma cell infiltration. Moreover, considering the reduced erythrocyte deformability that we observed in MM, it is not possible to exclude that RDW can indirectly provide with assertive information about this hemorheological parameter with a key role in the microcirculation district, very often involved in MM. Therefore, this study represents a starting point for subsequent analyses, possibly prospective and with a more numerous and homogeneously distributed sample, aimed at further defining the importance of RDW in multiple myeloma, considering also other important information at now missing, as the cytogenetic profile. This erythrocyte index could in fact represent an indirect parameter for the evaluation of multiple aspects, to make an increasingly complete assessment of patients.

## Methods

### Patients

We performed a single-centre retrospective analysis of patients with symptomatic MM, diagnosed and treated at the Hematology Division of the "Paolo Giaccone" University Hospital of Palermo. The diagnosis of symptomatic MM was made according to the revised International Myeloma Working Group criteria^61^, and specifically the patients were diagnosed with MM if they had 10% or more bone marrow clonal plasma cells or a biopsy-proven plasmacytoma, serum and/or urinary monoclonal protein (except in non-secretory patients), and the presence of one or more myeloma defining events (MDE). MDE consist of CRAB features (hypercalcemia, renal failure, anemia, or lytic bone lesions) as well as 3 specific biomarkers: clonal bone marrow plasma cells ≥ 60%, serum free light chain ratio ≥ 100 (provided involved FLC level is ≥ 100 mg/L), and more than one focal lesion on MRI. To estimate the sample size for our analysis, we considered the total number of patients who, on average, are studied in our division for suspected multiple myeloma, in a period of approximately 5 years (population size). Considering therefore the population size to be approximately 260 patients, assuming a 95% confidence interval and a margin of error of 5%, as a result, data from 190 were collected from January 1, 2017 to September 30, 2022, obtaining a representative sample of the study population. The inclusion criteria were: (1) age ≥ 18 years, (2) diagnosis of symptomatic multiple myeloma according to the above criteria, (3) subjects who have not had malignancy in the 6 months prior to enrolment or who, at most, have carcinoma in situ undergoing treatment with successful total eradication of the neoplasm. Patients with monoclonal IgM component, monoclonal gammopathy of undetermined significance (MGUS), smoldering multiple myeloma (SMM), and amyloidosis at the time of diagnosis were excluded. We also considered as exclusion criteria the presence of liver cirrhosis of any origin, inflammatory conditions including infections or collagen diseases, concomitant malignancy, and blood transfusion within the previous 3 months. The patients were staged according to the International Staging System (ISS)^62^, and their baseline demographics information were obtained from their medical records. All the informed consents have been obtained from the patients. The study was conducted in accordance with the Declaration of Helsinki and approved by the Ethics Committee of the University Hospital of Palermo (report N° 01/2022). Informed consent was obtained from all the enrolled subjects.

### Methods

The patients baseline laboratory data were obtained from blood tests performed at the central laboratory of our hospital, from samples collected at the time of initial diagnosis and before systematic treatment. Specifically, we evaluated serum β-2 microglobulin (B2M), serum calcium, serum creatinine, serum albumin, serum lactate dehydrogenase (LDH), bone marrow (BM) plasma cell infiltration, serum protein electrophoresis (SPE), hematocrit (Ht), hemoglobin expressed in gr/L, MCV expressed in fL and RDW. RDW is reported as a coefficient of variation (percentage) of red blood cell volume. The reference range for RDW in our institution is 11–15%.

### Statistical analysis

Statistical analysis was performed using GraphPAd Prism vers. 9.5. The data were expressed as medians and interquartile ranges. The one-way variance analysis, concerning the comparison between the different isotypes and between different stages of ISS, was performed with the Kruskal–Wallis test, integrated by the Dunn test. The median comparison was made with the Mann-Whitney test. The null hypothesis was evaluated for values of p ≤ 0.05.

### Supplementary Information

Supplementary Information.

## Data Availability

Correspondence and requests for materials should be addressed to M.C.
