# Supplementary material for: Changes in RDW according to prognostic predictors in newly diagnosed multiple myeloma
Source: Sci Rep. 2024 Feb 3;14:2832. doi: 10.1038/s41598-024-53385-6 (PMC10838310; doi:10.1038/s41598-024-53385-6)
Supplement: Supplementary file 1 — Supplementary Information. [file 41598_2024_53385_MOESM1_ESM.docx]

# Supplementary Information

# Title: Changes in RDW according to prognostic predictors in newly diagnosed multiple myeloma.

# Authors: Carlisi M^1^, Lo Presti R^2^, Plano F^3^, Mancuso S^4^, Siragusa S^5^, Caimi G^6^

1. *Department of Health Promotion, Mother and Child Care, Internal Medicine and Medical Specialties, University of Palermo, Palermo, Italy;* [*melaniacarlisi@yahoo.it*](mailto:melaniacarlisi@yahoo.it)*. ORCID: 0000-0002-5641-2974*
2. *Department of Psychology, Educational Science and Human Movement, University of Palermo, Palermo, Italy;* [*rosalia.lopresti@unipa.it*](mailto:rosalia.lopresti@unipa.it)*. ORCID: 0000-0002-7491-568X*
3. *Department of Health Promotion, Mother and Child Care, Internal Medicine and Medical Specialties, University of Palermo, Palermo, Italy;* [*salvatrice.mancuso@unipa.it*](mailto:salvatrice.mancuso@unipa.it)*. ORCID: 0000-0002-7926-090X*
4. *Department of Health Promotion, Mother and Child Care, Internal Medicine and Medical Specialties, University of Palermo, Palermo, Italy;* [*sergio.siragusa@unipa.it*](mailto:sergio.siragusa@unipa.it)*. ORCID: 0000-0002-1641-6508*
5. *Department of Health Promotion, Mother and Child Care, Internal Medicine and Medical Specialties, University of Palermo, Palermo, Italy;* [*gregorio.caimi@unipa.it*](mailto:gregorio.caimi@unipa.it)*. ORCID: 0000-0001-8964-255X.*

**Corresponding author**: Melania Carlisi, Department of Health Promotion, Mother and Child Care, Internal Medicine and Medical Specialties, University of Palermo, Via del Vespro 129, 90127, Palermo, Italy; Phone/Fax: +39 0916554410/0916554402; e-mail: [melaniacarlisi@yahoo.it](mailto:melaniacarlisi@yahoo.it).

**Supplementary Table 1**: Medians, IQRs and ranges of the erythrocyte parameters in MM patients

| **All MM (n=190)** | **Median (IQR)** | **Range** |
| --- | --- | --- |
| *Ht %* | 31.35 (9.05) | 21.00 - 46.70 |
| *Hb (g/dl)* | 10.30 (2.925) | 7.00 - 16.00 |
| *MCV (fl)* | 92.45 (8.82) | 65 - 110.9 |
| *RDW %* | 15.10 (3.60) | 12.10 - 23.40 |

IQR = interquartile range; MM = multiple myeloma; Ht = hematocrit; Hb = hemoglobin; MCV = mean cell volume; RDW = Red blood cells distribution width.

**Supplementary Table 2:** Medians of the prognostic factors in all and in subgroups of MM patients

| **Median** | **All MM (n=190)** | **LCMM**  **(n=27)** | **IgA MM**  **(n=56)** | **IgG MM (n=107)** |
| --- | --- | --- | --- | --- |
| Albumin (g/l) | 36.70 | 39.90 | 33.85 | 37.00 |
| Beta2-MG (mg/l) | 4.70 | 4.40 | 4.90 | 4.70 |
| LDH (U/L) | 172.5 | 179 | 152.5 | 179 |
| BMPC % | 40 | 40 | 60 | 30 |

MM = multiple myeloma; LCMM= Light chain multiple myeloma; Beta2-MG = Beta2-microglobulin; LDH = Lactate dehydrogenase; BMPC = bone marrow plasma cell.
